# Noncovalent n → π* Interactions in Collagen: The Key for Everlasting Bonds?

**DOI:** 10.1021/acscentsci.4c01691

**Published:** 2024-10-11

**Authors:** Marina Rubini

**Affiliations:** School of Chemistry, University College Dublin, Belfield, Dublin 4, Ireland

Collagen is the most abundant protein in mammals. It is a structural protein of the extracellular matrix and it is responsible for the stability of bones, muscles, and connectivity tissues in general, thus playing an essential role in human biology. Intact collagen fragments have been also discovered in dinosaur fossils that have been dated back to 195 million years, although the half-life of peptide bonds in aqueous solutions is “only” ∼500 years.^1^ The striking mechanical stability of collagen and its resistance toward proteolytic digestion and hydrolysis have been mainly attributed to its unique three-dimensional structure and the protection offered by bone mineralization, among others. In this issue of *ACS Central Science*, Raines and co-workers experimentally demonstrated that noncovalent n → π* interactions play an essential role in preventing hydrolysis, thus offering an explanation at the atomic level for the extreme longevity of collagen.^2^

Collagen is a fibrous protein with a remarkable quaternary structure, in which three left-handed helices are twisted together into a right-handed triple helix (Figure 1a).

**Figure 1:**
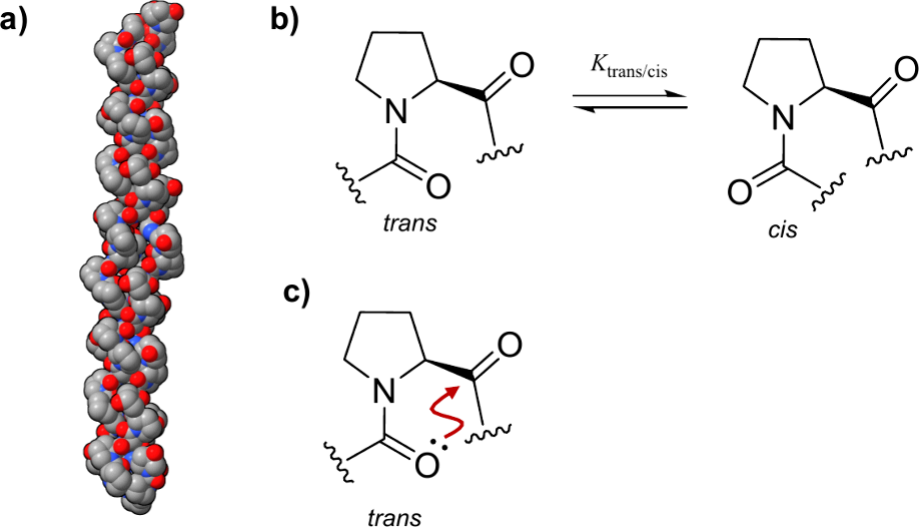
a) Crystal structure of a collagen-like peptide (PDB: 1CAG). The figure was prepared with ChimeraX. b) Proline peptide bond isomerization equilibrium. c) n → π* interactions increase the stability of the *trans* peptide bond by donating lone-pair electron density into the empty π* orbital of an adjacent carbonyl group.

The elegance and strength of the collagen structure are dictated by its primary structure that consists of a repeating Xaa-Yaa-Gly (Xaa and Yaa = any amino acid; Gly = glycine) sequence. The amino acids that occupy the Xaa and Yaa positions are often l-proline (Pro) and (4*R*)-hydroxyproline (Hyp), respectively, making Pro-Hyp-Gly the most common triplet in collagen.^3^ The small and flexible glycine residue is needed for enabling the tight packing of the individual helices within the triple helix, while Pro and Hyp help the preorganization of the individual strands in the correct conformation.^4^ The structure of collagen is further stabilized by interstrand hydrogen bonds within the triple helix. In the collagen structure, the location of the Xaa-Yaa and Yaa-Gly peptide bonds near the center of the helical axis makes them inaccessible to water and, therefore, resistant to hydrolysis, while the Gly-Xaa peptide bond is more exposed and thus represents the “weak” link in this formidable construction. However, other noncovalent interactions are involved in the increase of collagen stability: n → π* interactions. These are rather weak, but very abundant interactions in the backbones of proteins that arise from the donation of electron density from a lone pair (n) of the oxygen atom of a carbonyl group into the π* orbital of an adjacent carbonyl group.^5^ The great number of n → π* interactions can make up to nearly 10 kcal/mol for a 100-residue protein, thus underlying their contribution to protein folding and stability.^6^

The peptide bonds in a protein can adopt either a *cis* or a *trans* conformation (Figure 1b), with *trans* being by far the most represented (>99%), in order to avoid steric clashes. However, although the *trans* conformation is still preferred in the Xaa-Pro bond (Xaa: any amino acid), the occurrence of *cis* prolyl-peptide bonds is more frequent (∼6% of all prolines) in the context of protein tertiary structures, because the steric hindrance of the pyrrolidine ring of Pro cannot be fully eliminated by the adoption of the *trans* conformation.^7,8^ Despite the high proline content in collagen, all peptide bonds are found in the *trans* conformation, suggesting that a *cis-*prolyl peptide bond might jeopardize the integrity of the structure.

In this work, Raines and co-workers showed that the adoption of the *trans* conformation might be essential for the protection of the weaker Gly-Xaa bond in collagen from hydrolysis, as stabilizing n → π* interactions are possible only in the presence of *trans* peptide bonds, which allow a closer approach of the amide oxygen to the adjacent carbonyl group (Figure 1c). The simple and elegant explanation given by Raines and co-workers is based on the Pauli exclusion principle: orbitals that are already filled cannot be occupied, thus preventing access of nucleophilic water molecules to the carbonyl group that is the acceptor of an n → π* interaction.

To support their hypothesis, the authors tested the hydrolysis rate of model compounds, *N*-acylated-l-Pro *p*-nitrophenyl (*p*NP) esters, with different propensities for the *cis* of the *trans* conformation, as a proxy for the determination of the hydrolytic stability of a peptide bond. The prevalence of the *trans* over the *cis* conformation was manipulated by installing acyl groups with differing steric bulk. This resulted in esters with a higher preference for the *trans* conformation when Pro was *N*-acylated with the bulkier pivaloyl group than the smaller acetyl or formyl group. The hydrolysis rates for the model compounds were monitored by following the increase in the absorbance at 400 nm, after the release of *p*-nitrophenolate. The observed rates showed that the *N*-acylated proline esters with a higher populated *trans* conformation were much less prone to spontaneous hydrolysis. The calculated rate for the hydrolysis reaction proceeding from the compounds in the *cis* conformation is compatible with the proposed mechanism. This suggests that the ester carbons, although solvent exposed, are protected from nucleophilic attacks by the Pauli exclusion principle in the *trans* conformation, due to favorable n → π* interactions. Computational analyses and X-ray crystallographic data for the *N*-acetyl-Pro-pNP and *N*-pivaloyl-Pro-*p*NP esters further corroborated this hypothesis, as the data are in agreement both with the distance (O···C=O) and the angle required for n → π* interactions to take place.

Researchers have intensively tried to manipulate n → π* interactions in peptides and proteins for creating more robust scaffolds, but no link had previously been established between these interactions and protection from hydrolysis. This is perhaps not the only explanation for the extreme stability of collagen, but it is definitely a powerful one. It would be also interesting to test how this finding applies to other molecules and if it can be generalized. The outcomes of this study might find applications in materials, drug design, and tissue aging. Understanding the mechanisms that prevent or allow hydrolysis provides a powerful tool to create molecular switches to turn the process on or off. This knowledge could therefore guide the design of both long-lived and biodegradable materials, thus having a potential impact on environmental challenges.

## References

[ref1] Smith R. M.; Hansen D. E. The pH–rate profile for the hydrolysis of a peptide bond. J. Am. Chem. Soc. 1998, 120, 8910–8913. 10.1021/ja9804565.

[ref2] Yang J.; Kojasoy V.; Porter G. J.; Raines R. T. Pauli Exclusion by n→π* Interactions: Implications for Paleobiology. ACS Central Sci. 2024, 10.1021/acscentsci.4c00971. PMC11503490 39463835

[ref3] Ramshaw J. A. M.; Shah N. K.; Brodsky B. Gly-X-Y tripeptide frequencies in collagen: a context for host-guest triple-helical peptides. J. Struct. Biol. 1998, 122, 86–91. 10.1006/jsbi.1998.3977. 9724608

[ref4] Shoulders M. D.; Raines R. T. Collagen structure and stability. Annu. Rev. Biochem. 2009, 78, 929–958. 10.1146/annurev.biochem.77.032207.120833. 19344236 PMC2846778

[ref5] Newberry R. W.; Raines R. T. The n→π* Interaction. Acc. Chem. Res. 2017, 50, 1838–1846. 10.1021/acs.accounts.7b00121. 28735540 PMC5559721

[ref6] Bartlett G. J.; Choudhary A.; Raines R. T.; Woolfson D. N. n-->pi* interactions in proteins. Nat. Chem. Biol. 2010, 6, 615–620. 10.1038/nchembio.406. 20622857 PMC2921280

[ref7] Pal D.; Chakrabarti P. Cis peptide bonds in proteins: residues involved, their conformations, interactions and locations. J. Mol. Biol. 1999, 294, 271–288. 10.1006/jmbi.1999.3217. 10556045

[ref8] MacArthur M. W.; Thornton J. M. Influence of proline residues on protein conformation. J. Mol. Biol. 1991, 218, 397–412. 10.1016/0022-2836(91)90721-H. 2010917

